# Tissue distribution and transmission of Rift Valley fever phlebovirus in European Culex pipiens and Aedes albopictus mosquitoes following intrathoracic inoculation

**DOI:** 10.1099/jgv.0.002025

**Published:** 2024-09-20

**Authors:** Jaume Gardela, Karen Yautibug, Sandra Talavera, Enric Vidal, Catherine Cêtre Sossah, Nonito Pagès, Núria Busquets

**Affiliations:** 1 IRTA, Centre de Recerca en Sanitat Animal (CReSA, IRTA-UAB), Campus de la Universitat Autònoma de Barcelona, Bellaterra, Barcelona, Spain; 2 Unitat mixta d’Investigació IRTA-UAB en Sanitat Animal, Centre de Recerca en Sanitat Animal (CReSA), Campus de la Universitat Autònoma de Barcelona, 08193 Bellaterra, Barcelona, Spain; 3 ASTRE, University of Montpellier, CIRAD, INRAe, Montpellier, France; 4 CIRAD, UMR ASTRE, Montpellier Cedex 34398, France; 5 CIRAD, UMR ASTRE, Guadeloupe, France

**Keywords:** arbovirus, Europe, immunohistochemistry, mosquito-borne virus

## Abstract

Rift Valley fever virus (*Phlebovirus riftense*, RVFV) poses significant economic challenges, particularly in African nations, causing substantial livestock losses and severe haemorrhagic disease in humans. In Europe, the risk of RVFV transmission is deemed moderate due to the presence of competent vectors like *Culex pipiens* and *Aedes albopictus*, along with susceptible animal vertebrate hosts across member states. This study investigates RVFV infection dynamics in European mosquito populations, aiming to enhance our understanding of their vectorial capacity and virus transmission, which can be useful for future investigations to improve RVFV surveillance, control programmes, and preventive treatments. Intrathoracic inoculation of European *Cx. pipiens* and *Ae. albopictus* with an RVFV virulent strain (RVF 56/74) enabled the assessment of virus tissue distribution and transmission. Immunohistochemistry analyses revealed widespread RVFV infection in all analysable anatomical structures at 5 and 14 days post-inoculation. Notably, the ganglionic nervous system exhibited the highest detection of RVFV in both species. *Cx. pipiens* showed more frequently infected structures than *Ae. albopictus*, particularly in reproductive structures. The identification of an RVFV-positive egg follicle in *Cx. pipiens* hints at potential vertical transmission. Saliva analysis indicated a higher transmission potential in *Cx. pipiens* (71.4%) compared to *Ae. albopictus* (4.3%) at the early time point. This study offers the first description and comparison of RVFV tissue distribution in *Ae. albopictus* and *Cx. pipiens*, shedding light on the susceptibility of their nervous systems, which may alter mosquito behaviour, which is critical for virus transmission. Overall, enhancing our knowledge of viral infection within mosquitoes holds promise for future vector biology research and innovative approaches to mitigate RVFV transmission.

## Introduction

The Rift Valley fever virus (*Phlebovirus riftense*, RVFV) (order: Bunyavirales; family: *Phenuiviridae*; genus: *Phlebovirus*) is a pathogenic virus transmitted by mosquitoes that has a significant impact on livestock farming in Africa and the Arabian Peninsula [1], since RVFV affects domestic ruminants, resulting in significant livestock losses and high abortion rates during outbreaks. Humans can be infected with RVFV via mosquito bites as well as by direct contact with tissues and fluids from infected livestock. Most human infections cause self-limiting febrile illness, but 1–2% of the infections cause more serious disease, often with high mortality rates [2]. Therefore, RVF is one of the 117 WOAH (World Organization for Animal Health)-listed diseases that require mandatory notification [3]. RVFV has been identified as a bioterrorist threat and classified as a category A agent by the Centers for Disease Control and Prevention [4]. Despite the low entry risk in the European Union (EU) due to strict rules on animal imports, the EU’s potential for RVFV transmission has been categorized as moderate, considering the presence of RVFV-competent vectors and the full susceptibility of animal hosts in all EU member states [5].

RVFV is maintained through horizontal transmission between domestic animals, mainly ruminants, and mosquitoes during epizootics, which are associated with heavy rains that result in a significant increase of mosquito populations and infected livestock. Since its discovery in Kenya in 1930 [6], several blood-feeding arthropods have been implicated as RVFV vectors. RVFV has been isolated from over 53 mosquito species belonging to 8 genera of the family *Culicidae* in areas where epizootics have occurred [7].

During intervals of unfavourable conditions for vectors, when mosquito abundance is insufficient to sustain extensive horizontal transmission, the prevailing hypothesis suggests that RVFV may persist through vertical transmission via infected eggs [8]. Aedine eggs, in particular, can survive desiccation, with both embryo and virus remaining viable [8]. However, the proof for vertical transmission remains limited. Existing evidence includes virus isolation from *Aedes mcintoshi* mosquitoes reared from field-collected larvae [9], detection of RVFV in field-collected males and females of *Aedes vexans* and *Culex quinquefasciatus* [10], and antigen detection in mosquito chorionated eggs in *Ae. mcintoshi* [11]. Recently, evidence of vertical transmission has also been reported in a *Culex tarsalis* colony [12]. However, the role of the virus survival during inter-epizootic periods remains uncertain and requires further investigation.

A minimum of 47 mosquito species have been shown in the laboratory to be capable of spreading the virus through bite after oral exposure or intrathoracic inoculation [7]. More than ten of these are found in the Mediterranean Basin, including *Culex pipiens*, *Culex theileri*, *Aedes caspius*, *Ae. vexans*, *Aedes albopictus* and *Aedes detritus* [8,13,15], but their vectorial capacity has received little attention. Vectorial capacity, defined as the ability of a vector population to transmit pathogens, is influenced by several factors, such as vector competence, the pathogen’s extrinsic incubation period, vector biting rate, vector density, and the probability of vector daily survival [16]. It has traditionally been believed that arboviruses are not pathogenic in their arthropod vectors, with persistent infections that typically do not result in severe fitness defects, as mosquitoes have been considered to be tolerant to arbovirus infections [17]. However, several studies have reported some fitness costs of infection and interference with host-seeking behaviour in their vectors [18,20]. Other reports on RVFV infection in *Cx. pipiens* showed a reduced ability to refeed, reduced fecundity, and reduced survival [21,22], but the exact mechanisms for how the arboviruses affect the fitness of infected mosquitoes are still unclear. Therefore, RVFV infection within mosquito vectors needs to be characterized to better understand RVFV transmission by mosquitoes, which is crucial for developing adequate surveillance and control programmes as well as for improving preventive and prophylactic measures, such as vaccines.

*Cx. pipiens*, which was involved in the RVFV outbreak in Egypt in 1977 [23], together with *Ae. albopictus*, are widely distributed mosquito species in Southern Europe and are considered potential RVFV vectors. However, to date, only a few studies have examined the virus distribution in *Cx. pipiens* tissues by immunohistochemistry (IHC) or electron microscopy [24,27], and most of them focused on non-European *Cx. pipiens* strains. One study detected viral RNA in tissues of a *Cx. pipiens* strain from London using the RNAscope technique [26]. Additionally, limited data are available concerning *Ae. albopictus*’s potential role as vector-competent species [13,28]. Vertical transmission has been a focal point of many investigations performed on different species of *Aedes* [11,29]. Therefore, the goals of the present study were to: (1) identify the anatomical structures infected with RVFV within potential European mosquito vectors (*Cx. pipiens* and *Ae. albopictus*), using IHC to gain insights into nervous system infection and vertical transmission in particular, and (2) evaluate the salivary gland barrier for RVFV using IHC and viral titration of saliva to better characterize horizontal transmission and explore the potential use of infected mosquitoes in animal models for RVFV vaccine and antiviral development.

## Methods

### Virus production and titration

*Ae. albopictus* C6/36 cells were used for the propagation of the virus stocks at 28 °C with 5% CO_2_. Vero CCL-81 cells were used to titrate the virus at 37 °C with 5% CO_2_. Both cells were cultured in Dulbecco’s modified Eagle’s medium (DMEM; Gibco Life Technologies, MA, USA) supplemented with 2% foetal bovine serum (FBS; Gibco Life Technologies, MA, USA) and 1× antibiotic–antifungal solution (Gibco Life Technologies, MA, USA).

A virulent RVFV strain (RVF 56/74), kindly provided by Dr Alejandro Brun (CISA-INIA) and originally isolated from cattle in 1974 [30], was used for mosquito experimental infection. The passage history of the virus included three passages in chicken embryo-related cells, seven passages in Madin–Darby Bovine cells (MDBK) and two passages in baby hamster kidney fibroblast cells (BHK-21). Afterwards, RVF 56/74 was propagated in C6/36 cells at a multiplicity of infection of 0.1 and titrated in Vero CCL-81 cells as previously described [13].

### Mosquito rearing

A *Cx. pipiens* strain from Gavà (Catalonia, Spain, 2012) (comprising a mix of *Cx. pipiens* biotype pipiens, *Cx. pipiens* biotype molestus and *Cx. pipiens* biotype hybrid) and an *Ae. albopictus* strain from Sant Cugat del Vallès (Catalonia, Spain, 2005) were used for RVFV infection. Both mosquito colonies were reared in the laboratory under the following environmental conditions: 25 °C, 80% relative humidity and a photoperiod cycle of 12 : 12 h (light : dark), including two crepuscular cycles of 30 min to simulate dawn and dusk for *Cx. pipiens* species. Larvae were kept in plastic trays containing 750 ml of dechlorinated tap water, which was renewed three times per week, and fed *ad libitum* with fish pellets (Goldfish Sticks-TETRA, Melle, Germany). Upon reaching pupal stage, they were immediately transferred to insect cages (BugDorm-1 Insect Rearing Cage W30×D30×H30 cm, Mega View Science, Talchung, Taiwan ROC). Adult mosquitoes were provided with 10% sucrose solution *ad libitum*.

### Infection design and sampling

The experimental infections were carried out at Institut de Recerca i Tecnologia Agroalimentàries – Centre de Recerca en Sanitat Animal (IRTA-CReSA) biosecurity level 3 (BSL3) facilities.

Non-blood-fed female mosquitoes (*n*=69 *Cx*. *pipiens*, *n*=58 *Ae*. *albopictus*), 7–10 days old, were inoculated intrathoracically with RVFV (6.5 log_10_TCID_50_ ml^−1^) using a manual microinjector (Sutter Instrument, CA, USA). Briefly, females were anaesthetized with CO_2_ and the tip of a pulled capillary was gently introduced into the lateral side of the thorax between the scutum and the post-spiracular area, avoiding the direct contact with spiracles. The intrathoracic inoculation of the virus allows precise control of the virus dose delivered to each mosquito, crucial for standardizing the infection process and ensuring consistency across experimental samples and species comparisons. By controlling the infectious dose and route precisely, we could investigate factors influencing virus dissemination and transmission, such as mosquito species.

The inoculated females were kept alive at the same rearing conditions for 5 or 14 days post-inoculation (p.i.) to enable comparisons with previous studies [22,31] and ensure that there were two time points sufficiently spaced to track the infection’s progression within the mosquito. At these time points, mosquitoes were anaesthetized with CO_2_ and preserved in formalin for 48 h at BSL3 facilities before being processed in paraffin-embedded blocks for IHC analysis (Table 1). As a control group, individuals were inoculated with PBS and maintained under the same rearing conditions before being sacrificed between 7 and 15 days p.i. (*n*=6 *Cx*. *pipiens*, *n*=16 *Ae*. *albopictus*). For viral titration of saliva, legs and wings were dissected and saliva samples (Table 1) were collected by the capillary technique as described previously [13] in 1.5 ml tubes containing 193 µl of DMEM (Gibco Life Technologies, MA, USA), 2% FBS (Gibco Life Technologies, MA, USA). Legs after dissection were collected in 1.5 ml tubes containing 0.5 ml DMEM and 1× antibiotic–antifungal solution (Gibco Life Technologies, MA, USA) with 2 mm solid glass beads for viral isolation. All samples were stored at −80 °C until analysis.

**Table 1.** Number of Rift Valley fever virus infected mosquitoes for immunohistochemistry and viral titration

	*Culex pipiens*		*Aedes albopictus*	
	**5 days p.i.**	**14 days p.i.**	**5 days p.i.**	**14 days p.i.**
**Immunohistochemistry**	10	10	8	10
**Viral titration (legs and saliva**)	14	na	23	na

na, not available

### RVFV detection by IHC

Formalin-fixed mosquitoes were embedded into paraffin blocks including from three to five mosquitoes, cut into 3 µm paraffin consecutive sections, and rehydrated. Sections were either stained with haematoxylin–eosin or immunostained. For RVFV immunostaining, sections were incubated with 3% H_2_O_2_ in methanol for 30 min at room temperature for endogenous peroxidase inhibition. Sections were incubated with antigen retrieval solution (S16999, DAKO) for 20 min at 97 °C, followed by an incubation with 2% of bovine serum albumin in PBS–Tween-20 for 1 h at room temperature to block unspecific staining. The monoclonal antibody directed against the RVFV nucleocapsid (10H3-4E4-3D5, CIRAD) was used as the primary antibody at dilution 1/1,000 and incubated at 4 °C overnight. A peroxidase-labelled polymer conjugated to goat anti-mouse immunoglobulins (AK4001, DAKO) was used as a secondary antibody (50 µl/slide) and incubated for 45 min at room temperature followed by an incubation with 3,3′-diaminobenzidine tetrahydrochloride hydrate (Sigma-Aldrich) substrate and 0.05% H_2_O_2_ for 6 min. Paraffin-embedded liver tissue from an RVFV-infected lamb was used as a positive control [32]. Samples were examined with an optical microscope (MOTIC BA-410E).

### RVFV antigen semi-quantification in anatomical structures

The brown staining as a result of IHC using an RVFV-specific primary antibody confirmed the detection of RVF nucleocapsid in infected mosquito tissues. Negative controls were analysed and studied first to determine unspecific staining, e.g. chitin content or the presence of artefacts that may lead to misinterpretations [33]. Structures such as the pharynx and the pumping organ were excluded from the analysis because of the chitinous layer lining.

To assess the distribution of RVFV antigen in tissues, a semiquantitative scoring scheme from 0 to 3 was established: 0, no cells stained; 1, few cells stained; 2, many cells stained; and 3, all cells stained. Accordingly, all the sampled females received a score per anatomical structure from the consecutive sections. The present RVFV IHC study was mainly limited by the capture of the anatomical structures in the sections of a block that were available, explaining why the obtained scorings are asymmetrical.

### RVFV detection in cell cultures

Saliva samples were titrated on six-well plates using the plaque assay methodology in Vero CCL-81 cells. Briefly, confluent Vero CCL-81 cells were incubated with 35 µl of saliva samples in a total volume of 300 µl for 1 h at 37 °C with 5% CO_2_. After incubation, an agarose overlay (214 230, BD Difco, 1%) was applied to the cells and the plates were incubated for 7 days at 37 °C with 5% CO_2_. Viral titres from saliva samples were expressed as plaque-forming units per volume (p.f.u. ml^−1^).

Virus detection in mosquito legs was carried out directly in 96-well plates containing a Vero CCL-81 cell monolayer to obtain qualitative results (positive/negative results). Briefly, leg samples were homogenized at 30 Hz for 1 min using TissueLyser II (Qiagen GmbH, Hilden, Germany). Homogenized samples (30 µl) were added to each well of the 96-well plates and incubated for 1 h at 37 °C. After incubation, 150 µl of post-infection cell culture media was added to each well and the plates were incubated for 7 days at 37 °C with 5% CO_2_ until cytopathic effect observation.

### Statistical analyses

The mean and standard deviations of the RVFV antigen semi-quantification scoring were calculated using R Commander (GUI) version 4.0.3. The Mann–Whitney U test (significance level of alpha=0.05) was selected to analyse whether the variations in scoring identified were statistically significant (1) within a species at different days p.i. (intraspecies) and (2) between species (interspecies).

## Results

### Viral tissue distribution in intrathoracically inoculated *Cx. pipiens* and *Ae. albopictus* by IHC analysis

**Fig. 1.**
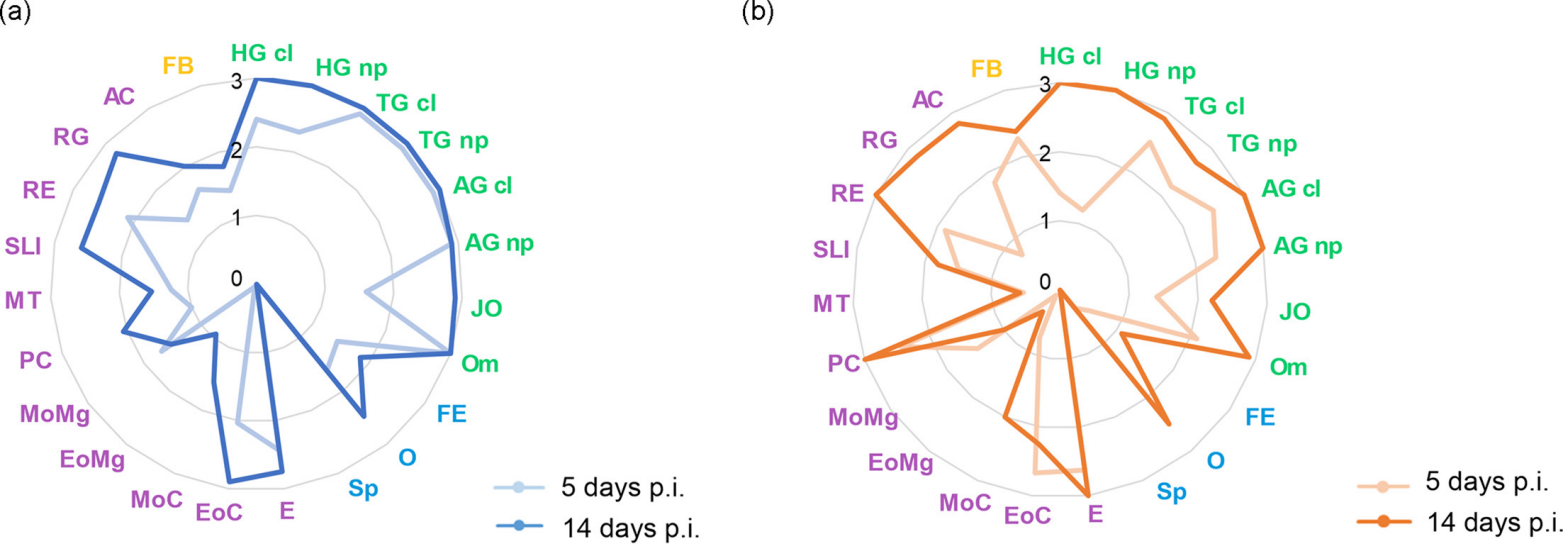
Rift Valley fever antigen mean semi-quantification scoring in tissues of (a) *Culex pipiens* and (b) *Aedes albopictus* at 5 and 14 days post-inoculation (p.i.). Nervous system in green: cortical layer (HG cl) and neuropile (HG np) of head ganglia, cortical layer (TG cl) and neuropile (TG np) of thoracic ganglia, cortical layer (AG cl) and neuropile (AG np) of abdominal ganglia, Johnston’s organ (JO), and ommatidia (Om). Reproductive system in blue: follicular epithelium (FE), oviducts (**O**), and spermathecae (Sp). Digestive system in purple: oesophagus (E), epithelium of the cardia (EoC), muscle of the cardia (MoC), epithelium of the midgut (EoMg), muscle of the midgut (MoMg), pyloric chamber (PC), Malpighian tubules (MT), small and large intestine (SLI), rectum epithelium (RE), rectum glands (RG), and acinar cells (AC). Intermediary metabolism and immune system in yellow: fat body (FB).

Presence of RVFV antigen was observed in all the anatomical structures that could be analysed in both species and at both studied time points (5 and 14 days p.i.), except for the spermathecae (Table 2). Most of the infected structures presented a high percentage of positivity in both studied mosquito species, although differences in the antigen intensity were observed (Fig. 1 and Table S1, available in the online version of this article). The structures of *Cx. pipiens* were more frequently infected compared to those of *Ae. albopictus*, especially in the digestive and reproductive systems. Low percentages of positivity in the midgut epithelium of both species indicated the low infection of the midgut from the haemocoel.

**Table 2.** Summary of Rift Valley fever virus antigen detection by immunohistochemistry and positivity percentage of infection at 5 and 14 days-post-infection in different anatomical structures of *Culex pipiens* and *Aedes albopictus*. Number of positive individuals/number of examined individuals (% of positivity)

		*Culex pipiens*				*Aedes albopictus*			
	**Anatomical structure**	**5 days p.i.**		**14 days p.i.**		**5 days p.i.**		**14 days p.i.**	
**Nervous system**	Head ganglia								
	Cortical layer	9/9	(100)	9/9	(100)	8/8	(100)	8/8	(100)
	Neuropile	9/9	(100)	9/9	(100)	8/8	(100)	8/8	(100)
	Thoracic ganglia								
	Cortical layer	9/9	(100)	9/9	(100)	6/6	(100)	9/9	(100)
	Neuropile	9/9	(100)	9/9	(100)	6/6	(100)	9/9	(100)
	Abdominal ganglia								
	Cortical layer	8/8	(100)	7/7	(100)	4/4	(100)	4/4	(100)
	Neuropile	7/7	(100)	7/7	(100)	4/4	(100)	4/4	(100)
	Johnston’s organ	9/9	(100)	8/8	(100)	7/7	(100)	7/7	(100)
	Ommatidia	9/9	(100)	9/9	(100)	8/8	(100)	8/8	(100)
**Reproductive system**	Follicular epithelium	10/10	(100)	10/10	(100)	6/8	(75)	6/6	(100)
	Undeveloped egg follicles	nq		nq		nq		nq	
	Developed egg follicles	1/9	(11)	0/8	(0)	na		na	
	Oviducts	7/7	(100)	7/7	(100)	1/3	(33)	4/4	(100)
	Spermathecae	0/6	(0)	0/3	(0)	0/3	(0)	0/7	(0)
**Digestive system**	Oesophagus	8/8	(100)	8/8	(100)	4/4	(100)	2/2	(100)
	Epithelium of the cardia	9/9	(100)	10/10	(100)	3/3	(100)	4/4	(100)
	Muscle of the cardia	1/8	(13)	8/9	(89)	2/2	(100)	1/1	(100)
	Epithelium of the midgut	1/9	(11)	9/10	(90)	1/7	(14)	2/5	(40)
	Muscle of the midgut	9/9	(100)	10/10	(100)	7/7	(100)	4/4	(100)
	Pyloric chamber	3/4	(75)	6/6	(100)	3/3	(100)	2/2	(100)
	Malpighian tubules	10/10	(100)	9/9	(100)	7/8	(88)	5/7	(71)
	Small and large intestine	9/9	(100)	8/8	(100)	3/3	(100)	3/5	(60)
	Rectum epithelium	9/9	(100)	6/6	(100)	4/4	(100)	4/4	(100)
	Rectum glands	8/8	(100)	6/6	(100)	2/4	(50)	3/3	(100)
	Apical cavities	nq		nq		nq		nq	
	Acinar cells	10/10	(100)	10/10	(100)	6/6	(100)	7/7	(100)
**Intermediary metabolism and immune system**	Fat body	10/10	(100)	9/10	(90)	8/8	(100)	10/10	(100)

na, not available; NQ, not quantifiable

In terms of antigen distribution, the ganglionic nervous system was the anatomical structure that presented the highest RVFV antigen immunolabelling intensity in both mosquito species (Fig. 1), even at the first studied time point (5 days p.i.). However, in *Ae. albopictus* most of the structures of the digestive system (e.g. muscle of the cardia, the midgut, the Malpighian tubules, and the rectum glands) and all the structures in the reproductive system showed lower RVFV antigen levels (<1) at this early time point (Fig. 1). The cortical layer and neuropile of the abdominal ganglia, the cortical layer of the thoracic ganglia and the pylorus depicted similar immunolabelling without statistical differences in the evolution of the antigen distribution in time or between species. The rest of the anatomical structures with statistical differences between both species and/or time points are detailed hereafter.

#### Nervous system

**Fig. 2.**
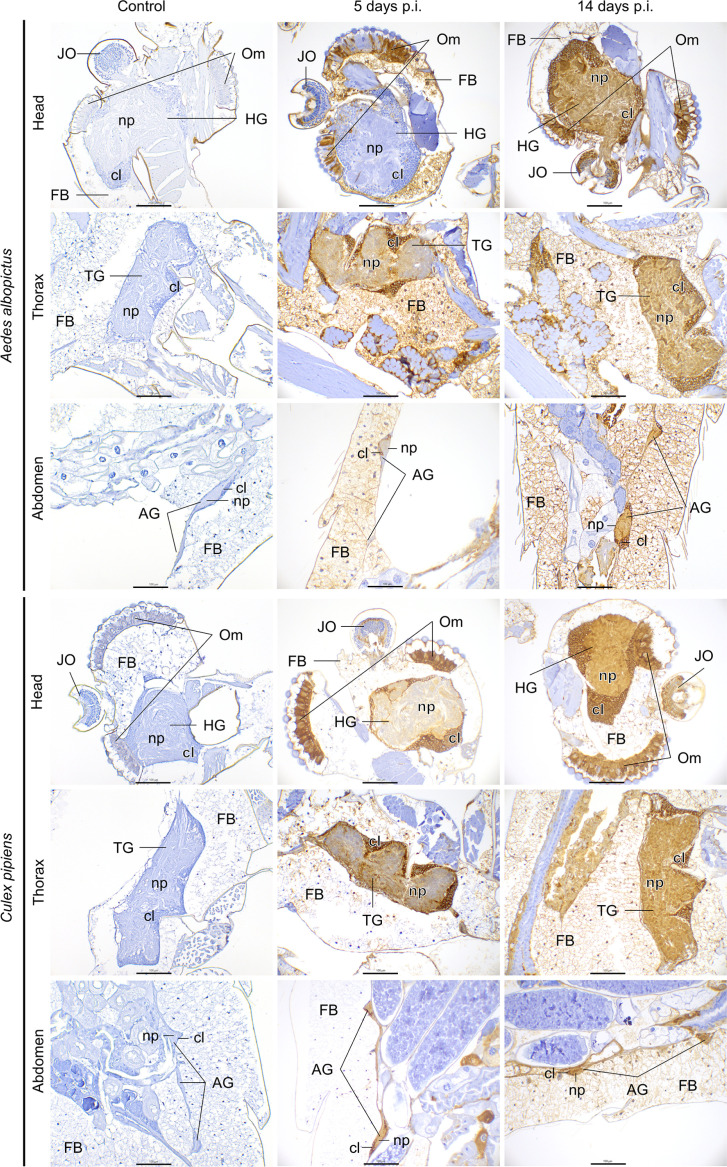
Immunohistochemistry examination of the nervous system and associated organs of *Aedes albopictus* and *Culex pipiens* mosquitoes of uninfected (control) and RVFV-infected mosquitoes at 5 and 14 days post-inoculation. Head ganglia (HG), thoracic ganglia (TG), abdominal ganglia (AG), cortical layer (cl), neuropile (np), Johnston’s organ (JO), ommatidia (Om), fat body (FB). Scale bar, 100 µm.

The ganglionic system showed the highest RVFV positivity in both mosquito species. The cortical layer and neuropile of the thoracic and abdominal ganglia showed similar RVFV intensity over time (Figs1  2), whereas the cortical layer and neuropile of the head ganglia showed higher antigen intensity at 14 days p.i. in *Cx. pipiens* (*P*_cortical layer_=0.0001; *P*_neuropile_=0.0015) and *Ae. albopictus* (*P*_cortical layer_=0.0004; *P*_neuropile_=0.0004). Notably, the head ganglia showed lower RVFV positivity in *Ae. albopictus* compared to *Cx. pipiens* at 5 days p.i. (*P*_cortical layer_=0.00197; *P*_neuropile_=0.0051). Additionally, the neuropile of the thoracic ganglia showed higher RVFV positivity in *Cx. pipiens* compared to *Ae. albopictus* at 5 days p.i. (*P*=0.005) and at 14 days p.i. (*P*=0.028) (Fig. 1). In all three ganglia the cortical layer consistently showed more antigen intensity than the neuropile (Fig. 2).

The Johnston’s organs from all individuals from both mosquito species were RVFV antigen-positive as early as 5 days p.i., and the antigen levels increased in intensity over time in both species (*P_Cx.pipiens_*=0.0003; *P_Ae.albopictus_*=0.009) (Fig. 2). The Johnston’s organ of *Cx. pipiens* showed a higher antigen intensity compared to *Ae. albopictus* at 14 days p.i. (*P*=0.003).

The evaluation of the ommatidia indicated high RVFV antigen intensity in both mosquito species at 5 days p.i. (Figs1  2). However, significant differences were observed between both species at this time point (*P*=0.0002). This suggests that ommatidia of *Ae. albopictus* required additional time to reach the same level of infection as *Cx. pipiens*, which reach a high RVFV antigen intensity at the first time point evaluated. The lenses of ommatidia were consistently free of antigen labelling throughout the study.

#### Reproductive system

Both mosquito species showed RVFV immunostaining in the follicular epithelium, albeit at relatively moderate/low intensity (Fig. 1). In particular, *Cx. pipiens* displayed significantly higher RVFV antigen intensity in the follicular epithelium compared to *Ae. albopictus* at both 5 days p.i. (*P*=0.0019) and 14 days p.i. (*P*=0.0049) (Figs1  3). Furthermore, a significant difference in antigen intensity was observed when comparing time points within *Ae. albopictus* (*P*=0.0241) and *Cx. pipiens* (*P*=0.015).

**Fig. 3.**
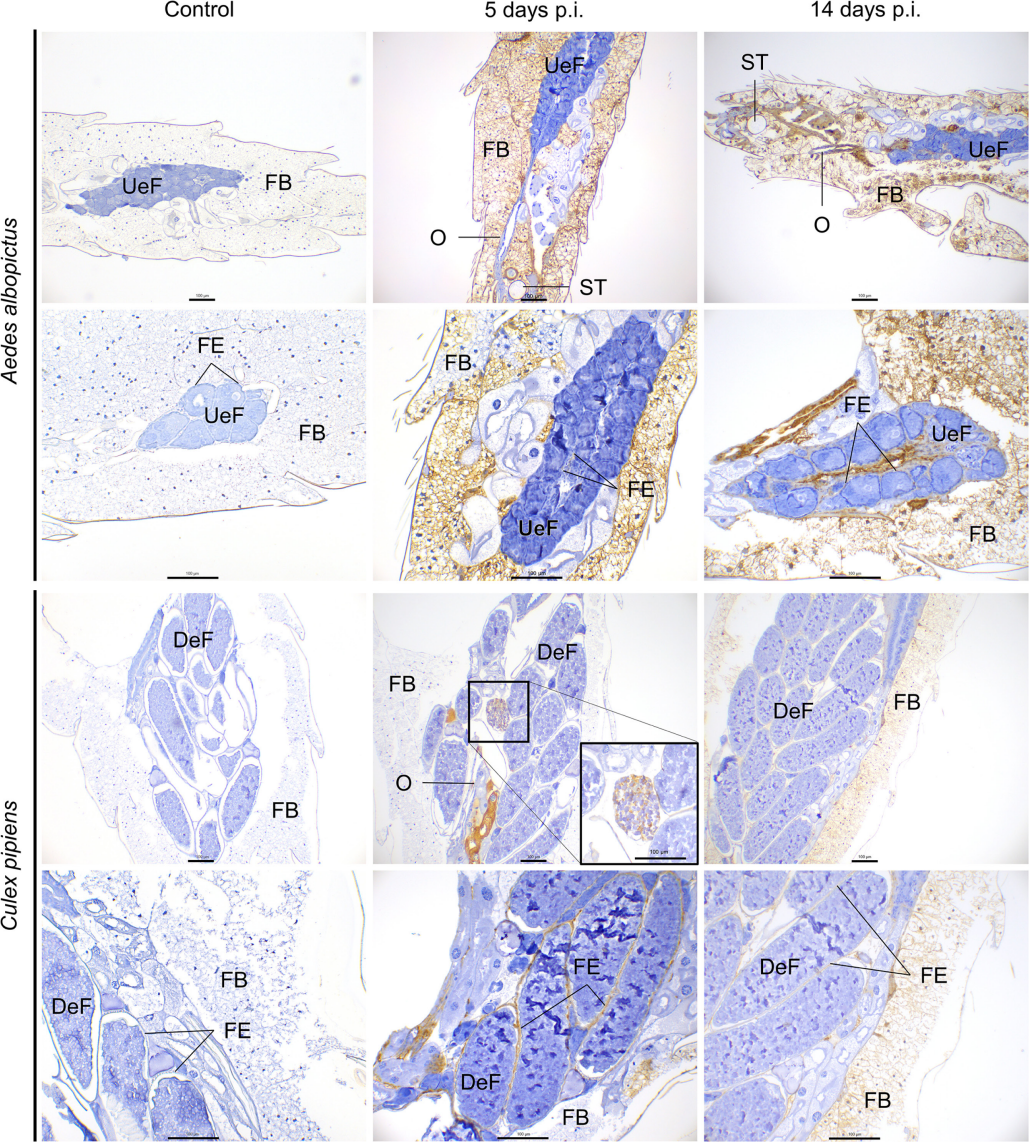
Immunohistochemistry examination of the reproductive system and associated organs of *Aedes albopictus* and *Culex pipiens* mosquitoes of uninfected (control) and RVFV-infected mosquitoes at 5 and 14 days post-inoculation. Undeveloped egg follicles (UeF), developed egg follicles (DeF), follicular epithelium (FE), oviduct (O), spermatheca (ST), fat body (FB). Scale bar, 100 µm.

Similar to the follicular epithelium, a higher RVFV antigen intensity was observed in the oviducts of *Cx. pipiens* compared to *Ae. albopictus* at 5 days p.i. (*P*=0.02). Although this difference was statistically significant at 5 days p.i., it became non-significant at 14 days p.i. when the antigen intensity was high in both species due to RVFV antigen intensity increasing over time (*P_Cx.pipiens_*=0.03; *P_Ae.albopictus_*=0.029).

*Cx. pipiens* showed evidence of autogeny in 89% of the females used in this study. The RVFV antigen levels in undeveloped egg follicles in the ovaries of both mosquito species was not quantifiable due to imprecise antigen labelling caused by weak signals. Remarkably, one developed egg follicle tested positive for RVFV at 5 days p.i. in *Cx. pipiens* (Fig. 3).

#### Digestive system

There were notable variations in RVFV immunolabelling across the digestive system, encompassing foregut, midgut, and hindgut tissues. The anatomical structures of the foregut (oesophagus and cardia) and hindgut (pyloric chamber, small and large intestine, rectum, and rectum glands) showed moderately higher infectivity, characterized by elevated positive percentages and average intensity scoring, compared to structures within the midgut for both mosquito species. Moreover, the foregut presented higher immunolabelling than the hindgut (Table 2 and Fig. 1).

**Fig. 4.**
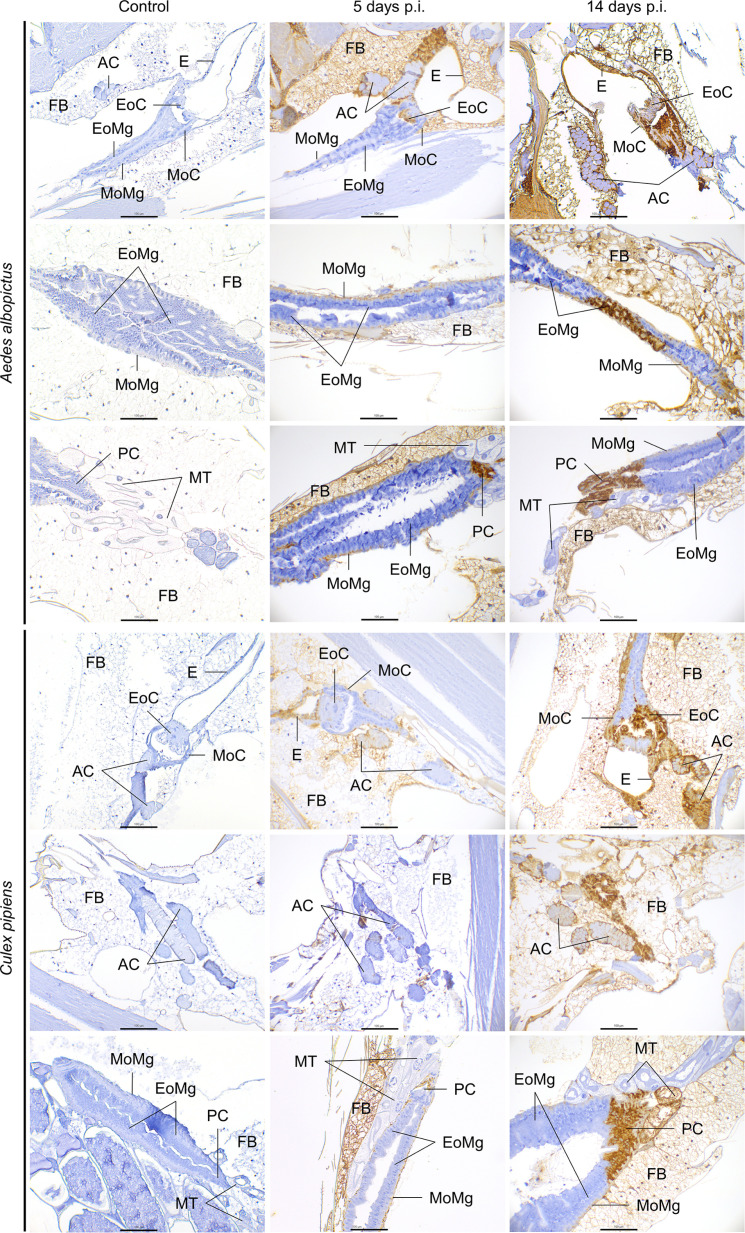
Immunohistochemistry examination of the digestive system and associated organs of *Aedes albopictus* and *Culex pipiens* mosquitoes of uninfected (control) and RVFV-infected mosquitoes at 5 and 14 days post-inoculation. Oesophagus (E), epithelium of the cardia (EoC), muscle of the cardia (MoC), epithelium of the midgut (EoMg), muscle of the midgut (MoMg), pyloric chamber (PC), acinar cells (AC), Malpighian tubules (MT), fat body (FB). Scale bar, 100 µm.

In all evaluated mosquito specimens, the muscle of the midgut was immunolabelled at both analysed time points (Table 2), although with relatively low intensity scoring (<2). Interestingly, the RVFV antigen intensity in the muscle of the midgut in *Ae. albopictus* decreased over time (*P*=0.039). However, in the midgut epithelium, a low positivity ratio (<15%) was observed at 5 days p.i., and RVFV detection increased over time for *Cx. pipiens* (*P*=0.0004). Nonetheless, the average intensity scoring remained low (<1), with only a few infected cells. In contrast, the cardia, marking the start of the midgut and the end of the foregut, showed significant immunolabelling to the virus, with 100% of the mosquitoes with positive immunolabelling and high intensity scoring (>2) in its epithelium, regardless of the days p.i. (Fig. 4). The muscle of the cardia showed an escalating RVFV infection over time in *Cx. pipiens* (*P*=0.001). This increase over time was also present in the oesophagus of the *Cx. pipiens* species (*P*=0.048). The pyloric chamber, where the midgut ends and hindgut begins, showed infected cells (Fig. 4) with a high positivity ratio (>75%) (Table 2), although only three individuals of *Ae. albopictus* could be evaluated.

Concerning Malpighian tubules, RVFV positivity was identified in most of the studied individuals, although with moderate (≤1.5) and low (≤0.6) antigen intensity for *Cx. pipiens* and *Ae. albopictus*, respectively (Fig. 1). The difference in viral labelling between species was significant at both days p.i. (*P*_5dpi_=0.0083; *P*_14dpi_=0.0054), with higher RVFV antigen intensity in *Cx. pipiens*.

Regarding the salivary glands, all individuals from both mosquito species presented infection in the acinar cells (Fig. 4). Significant differences were observed between species at 14 days p.i. (*P*=0.0059), with high scoring particularly evident in *Ae. albopictus*. Moreover, antigen intensity increased over time in both species, with a significant difference from 5 to 14 days p.i. in *Ae. albopictus* (*P*=0.0022). The apical cavities of both species showed weak brown staining inside, making quantification of RVFV challenging.

#### Fat body

The examination of the fat body revealed a higher RVFV antigen intensity in *Ae. albopictus* compared to *Cx. pipiens* at 5 days p.i. (*P*=0.0025) (Figs2^4^). Once the virus entered the fat body, it was not cleared, and RVFV positivity persisted in both species over time.

### Viral detection in legs and saliva of intrathoracically inoculated *Cx. pipiens* and *Ae. albopictus* by viral titration

All *Cx. pipiens* and *Ae. albopictus* legs and wings analysed were positive for RVFV by viral isolation.

**Fig. 5.**
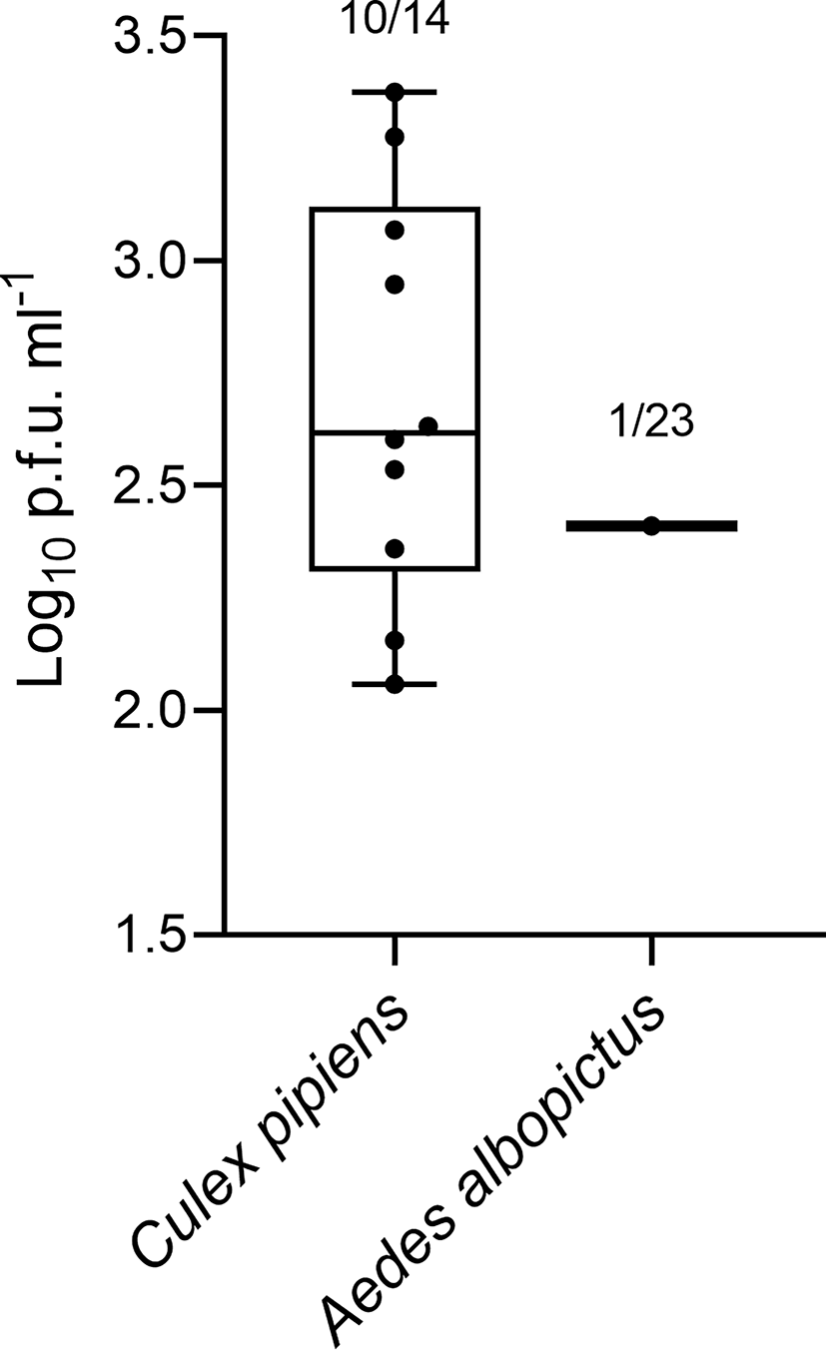
Rift Valley fever virus titre in saliva from *Culex pipiens* and *Aedes albopictus* at 5 days p.i. [log_10_ plaque-forming units (p.f.u.) ml^−1^].

The virus was successfully isolated from saliva in both mosquito species at 5 days p.i., with a higher isolation rate in *Cx. pipiens* (71.4%, 10/14) compared to *Ae. albopictus* (4.3%, 1/23). Despite the differences in isolation, the viral titres were similar among mosquitoes with positive saliva, ranging from 2.06 to 3.38 log_10_ p.f.u. ml^−1^ (Fig. 5).

Unfortunately, no results were obtained for saliva collected at 14 days p.i. due to bacterial contamination of the samples.

## Discussion

Following intrathoracic inoculation of RVFV, disseminated immunolabelling was observed in all females of both studied mosquito species, *Cx. pipiens* and *Ae. albopictus*, indicating a successfully disseminated infection. This outcome was anticipated, given that intrathoracic inoculation effectively bypasses the midgut barrier. Intrathoracic inoculation was used to ensure consistent and efficient infection, although infecting mosquitoes via an infected blood meal might have provided a more representative model of natural infection dynamics. This approach could have highlighted potential differences in the efficiency of virus passage across the midgut barrier between the two mosquito species used and, in turn, could influence the reported transmission potential through saliva analysis. While the dissemination of infection had been previously analysed for other *Cx. pipiens* populations and various virulent RVFV strains [22,27], this investigation is, to the best of our knowledge, the first assessment of RVFV dissemination in intrathoracically inoculated *Ae. albopictus*.

The rapid dissemination of RVFV infection was evidenced in both mosquito species, showing infection in most anatomical structures as early as the first analysed time point (5 days p.i.). This observation agrees with the high levels of viral replication reported in the initial days after intrathoracic inoculation in *Cx. pipiens* [22]. However, in contrast to this previous study, which found that all inoculated *Cx. pipiens* mosquitoes transmitted the virus, our study revealed the presence of a salivary gland infection barrier in both mosquito species tested. However, the salivary gland infection barrier was more pronounced in *Ae. albopictus* than in *Cx. pipiens*. This finding aligns with previous vector competence studies, particularly those involving oral infections [13]. The high transmission potential observed by virus isolation from saliva samples at 5 days p.i. in *Cx. pipiens* supports that this European species may function as a competent vector for RVFV as previously pointed out [13]. In fact, *Cx. pipiens* was previously implicated as the potential vector in the first reported outbreak of RVFV in Egypt, outside of sub-Saharan Africa [23]. The transmission potential reported in *Cx. pipiens* compared to *Ae. albopictus* suggests that *Cx. pipiens* is a prospective mosquito species that might be useful for vaccine development studies to mimic natural RVFV challenges in animals. However, such studies should consider using a more recent RVFV strain to avoid potential genetic instability and reassortment due to multiple passages in the strain used in this study that could alter our results following mosquito infection.

The nervous system was one of the early infected anatomical structures in both species that showed high immunolabelling at 5 days p.i. The antigen distribution observed in both mosquito species, with a higher presence in the cortical layer than in the neuropile, aligns with findings from a previous study involving a different *Cx. pipiens* population and another virulent RVFV strain [25]. The observed significant susceptibility of the nervous system to RVFV infection could potentially alter the behaviour of infected females, subsequently affecting the vectorial capacity. Nevertheless, whether these alterations increase or diminish vectorial capacity remains an unanswered question.

Regarding the reproductive system, our results indicate a higher susceptibility to virus infection in the reproductive organs in *Cx. pipiens* compared to *Ae. albopictus*. Specifically, our IHC analysis revealed 100% positivity in the follicular epithelium and the oviducts of *Cx. pipiens*, with one developed egg follicle testing positive at 5 days p.i. over nine infected females displaying developed egg follicles (11.1%). These results align with previous research conducted in *Ae. mcintoshi*, in which one RVFV positive egg was observed in 8.6% (3/35) of the infected females [11]. However, our findings contrast starkly with a previous report on *Cx. pipiens* infected with RVFV [25], where positivity was restricted to the oviducts and, crucially, no evidence for infection was detected inside egg follicles (either developed or undeveloped). Of note, the detection of one positive developed egg follicle is consistent with an investigation in *Cx. tarsalis* infected with RVFV, where ovaries, egg rafts, and progeny were found to be infected [12]. Our results for *Ae. albopictus* are in line with previous research on *Ae. aegypti* and RVFV, which detected antigen positivity in the ovariole sheath and the follicular epithelium but not in egg follicles [29], but contrast with RVFV-positive results in developed egg follicles in *Ae. mcintoshi* [11]. These findings raise questions about the role of *Culex* species in vertical transmission, a phenomenon traditionally attributed to *Aedes* species. Our results suggest that transovarial transmission may be more likely to occur in *Cx. pipiens*, having potentially significant epidemiological implications in endemic maintenance of RVFV by vertical transmission. It is crucial to emphasize that the variations in results could stem from the use of different viral strains, mosquito populations, and methodologies in the studies mentioned. Therefore, further studies, including blood-fed mosquitoes and a larger sample size, are essential to observe developed egg follicles in *Ae. albopictus* and confirm viable vertical transmission in *Cx. pipiens*.

In the context of the digestive system, our study reveals distinct infection patterns among several digestive tissues of both mosquito species. The midgut showed lower infection rates than the foregut and hindgut tissues. When comparing the foregut and hindgut, the foregut, which includes the cardia, showed higher susceptibility. These observations align with findings from a previous IHC study involving RVFV intrathoracically inoculated in *Cx. pipiens* [25].

Despite not evaluating the midgut as distinct anterior and posterior portions, our study showed RVFV positivity just after the end of the cardia or just before the beginning of the pyloric chamber. Therefore, in both studied mosquito species, our results suggest that RVFV infected the cardia and the pyloric chamber rapidly, with virions progressively reaching the midgut. This supports the role of the cardia as a viral amplifier in the digestive system [24,34]. Regarding the posterior midgut, the pyloric chamber showed a similar infection pattern to the cardia, showcasing early and high virus infection. The viral spread from the haemocoel into the midgut via the pyloric chamber may also apply to Malpighian tubules, which are open-ended near to the pyloric chamber [35]. This study marks the first evidence of RVFV infection in Malpighian tubules in two mosquito species. This is a significant advancement, as a previous IHC study on *Cx. pipiens* infected with RVFV was unable to assess this particular structure due to nonspecific staining [25]. Further studies are needed to investigate the effects of RVFV infection on the renal excretory system, considering its importance in mosquito osmoregulatory function.

RVFV could potentially access the digestive system through the tracheae system from the haemocoel [27,36]. Similar viral routes may be involved in salivary gland infection, as evidenced in our study by RVFV infection in acinar cells. Although both species showed similar susceptibility to the virus at the early time point evaluated by IHC, viral isolation from saliva indicated a stronger salivary gland escape barrier for *Ae. albopictus* than for *Cx. pipiens* at 5 days p.i. This aligns with vector competence results reported for both mosquito species [13]. Unfortunately, viral isolation from saliva could not be evaluated at 14 days p.i., emphasizing the need for further research to elucidate the dynamics of RVFV infection in mosquito salivary glands and transmission to new hosts, essential for developing effective intervention strategies.

Overall, the anatomical structures of *Cx. pipiens* consistently showed an earlier and more intense infection compared to *Ae. albopictus*, a pattern notably divergent from the observed trend in the fat body. The fat body plays a crucial role in metabolizing several substances that are subsequently released into the circulatory system [37]. Previous research has reported the infection of the fat body with different vector–arbovirus combinations [25,29, 38, 39]. The intriguing aspect arises from the apparent contradiction between the higher infection of the fat body in *Ae. albopictus* and the earlier and more intense infection observed in other anatomical structures of *Cx. pipiens*. Whether the intense infection of the fat body in *Ae. albopictus* is linked to a lower infection in other anatomical structures, or, conversely, if the intense infection of other structures in *Cx. pipiens* affects the fat body differently, is a question that demands a more in-depth and comprehensive investigation.

## Conclusions

The outcomes of the present study highlight the susceptibility of both European *Cx. pipiens* and *Ae. albopictus* to RVFV, with a high number of anatomical structures infected. It is worth noting that these mosquito species show widespread RVFV infection in the nervous system, which might suggest a potential impact on mosquito behaviour. Additionally, the identification of a positive egg follicle in *Cx. pipiens* suggests the potential for this mosquito species not only to maintain RVFV horizontally, as traditionally associated with *Culex* species, but also vertically. It is crucial to acknowledge that no studies on vector competence in infected progeny have been conducted to date. Therefore, investigating this topic in more detail may lead to new avenues for research.

To sum up, enhancing our fundamental understanding of the specific anatomical structures of viral infection within mosquitoes paves the way for future research into mosquito behaviour and the potential for vertical transmission of RVFV.

## supplementary material

10.1099/jgv.0.002025 Uncited Table S1.

## Abbreviations

BHK-21: baby hamster kidney fibroblast cells
BSL3: biosecurity level 3
days p.i.: days post-inoculation
DMEM: Dulbecco’s modified Eagle’s medium
EU: European Union
FBS: foetal bovine serum
IHC: immunohistochemistry
IRTA-CReSA: Institut de Recerca i Tecnologia Agroalimentàries – Centre de Recerca en Sanitat Animal
MDBK: Madin-Darby Bovine cells
p.f.u.: plaque forming units
RVFV: Rift Valley fever virus
WOAH: World Organization for Animal Health
