# Supplementary material for: Tissue distribution and transmission of Rift Valley fever phlebovirus in European Culex pipiens and Aedes albopictus mosquitoes following intrathoracic inoculation
Source: J Gen Virol. 2024 Sep 20;105(9):002025. doi: 10.1099/jgv.0.002025 (PMC11649246; doi:10.1099/jgv.0.002025)
Supplement: Uncited Table S1. [file jgv-105-02025-s001.pdf]

Supplementary Table 1. Summary of Rift Valley fever virus antigen semiquantitative scoring system at 5- and 14-days-post-infection (mean ± SD) in different anatomical structures of *Culex pipiens* and *Aedes albopictus* and summary of p-values of the analyses including variations in scoring system between species (interspecific) and within a species at different days-post-infection (intraspecific).

	Culex pipiens				*Aedes albopictus*				Interspecific		Intraspecific	
	5dpi		14 dpi		5dpi		14 dpi				*Cx. pipiens*	*Ae. albopictus*
	Mean	SD	Mean	SD	Mean	SD	Mean	SD	5 dpi	14 dpi	5 *vs.* 14 dpi	
Head ganglia												
cortical layer	2.45	0.37	3.00	0.00	1.45	0.41	2.98	0.07	<0.01	0.29	<0.001	<0.001
neuropile	2.31	0.64	3.00	0.00	1.24	0.50	2.98	0.07	<0.01	0.29	<0.01	<0.001
Thoracic ganglia												
cortical layer	2.92	0.24	3.00	0.00	2.54	0.47	2.89	0.31	0.08	0.32	0.32	0.17
neuropile	2.92	0.24	3.00	0.00	2.15	0.52	2.70	0.41	<0.01	0.03	0.32	0.06
Abdominal ganglia												
cortical layer	2.94	0.17	3.00	0.00	2.50	0.50	3.00	0.00	0.12	NA	0.35	0.13
neuropile	2.86	0.35	2.86	0.35	2.29	0.71	3.00	0.00	0.15	0.45	1.00	0.13
Johnston's organ	1.56	0.38	2.94	0.17	1.38	0.36	2.17	0.43	0.38	<0.01	<0.001	<0.01
Ommatidia	2.97	0.09	2.96	0.10	2.08	0.29	2.91	0.17	<0.001	0.47	-	-
Follicular epithelium	1.40	0.34	1.93	0.47	0.52	0.47	1.09	0.15	<0.01	<0.01	<0.05	<0.05
Undeveloped egg follicles	NQ	NQ	NQ	NQ	NQ	NQ	NQ	NQ	NQ	NQ	-	-
Developed egg follicles	0.02	0.06	0.00	0.00	NA	NA	NA	NA			-	-
Oviducts	1.62	0.36	2.48	0.66	0.33	0.47	2.50	0.50	<0.05	1.00	<0.05	<0.05
Spermathecae	0.00	0.00	0.00	0.00	0.00	0.00	0.00	0.00	NA	NA	-	-
Esophagus	2.44	0.42	2.75	0.66	2.63	0.41	3.00	0.00	0.48	0.62	<0.05	0.27
Epithelium of the cardia	2.04	0.74	2.90	0.30	2.67	0.47	2.25	0.83	0.21	0.09	<0.01	0.55
Muscle of the cardia	0.06	0.17	1.56	0.83	0.75	0.25	2.00	0.00	<0.05	0.57	<0.01	0.67
Epithelium of the midgut	0.06	0.16	0.93	0.47	0.10	0.23	0.40	0.49	0.78	0.12	<0.001	0.24
Muscle of the midgut	1.69	0.42	1.52	0.50	1.46	0.38	1.00	0.00	0.30	0.06	0.40	<0.05
Pyloric chamber	1.00	1.17	2.06	0.66	2.44	0.42	3.00	0.00	0.21	0.09	0.16	0.20
Malpighian tubules	1.24	0.65	1.53	0.60	0.53	0.34	0.58	0.43	<0.01	<0.01	0.19	0.86
Small and large intestine	1.50	0.42	2.60	0.42	1.50	0.41	1.80	1.47	1.00	0.75	<0.01	0.64
Rectum epithelium	2.11	0.39	2.58	0.61	1.88	0.74	3.00	0.00	0.40	0.22	0.14	<0.05
Rectum glands	1.38	0.54	2.79	0.37	0.75	0.83	2.83	0.24	0.22	1.00	<0.01	<0.05
Apical cavities	NQ	NQ	NQ	NQ	NQ	NQ	NQ	NQ	NQ	NQ	-	-
Acinar cells	1.62	0.66	2.01	0.66	1.82	0.19	2.82	0.21	0.41	<0.01	0.07	<0.01
Fat body	1.42	0.46	1.78	0.85	2.28	0.42	2.38	0.57	<0.01	0.16	0.22	0.89

NQ: not quantifiable. NA: not available.
